# The value of real-time shear wave elastography in spontaneous preterm birth

**DOI:** 10.1097/MD.0000000000039288

**Published:** 2024-08-16

**Authors:** Huiling Lu, Yang Liu, Fangrui Yang, Dan Wu, Jiarui Qi, Yonghao Ji, Mengli Hu

**Affiliations:** a Department of Ultrasonography, Baoji Central Hospital, Shaanxi, China; b Department of Gynecology, Baoji Central Hospital, Shaanxi, China.

**Keywords:** cervical length, shear wave elastography, spontaneous preterm birth, uterocervical angle

## Abstract

This study aimed to investigate the predictive value of real-time shear wave elastography (SWE) for spontaneous preterm birth (SPB). This study prospectively selected 175 women with singleton pregnancies at 16 to 36 weeks of gestation. Cervical length (CL) and uterocervical angle (UCA) were measured using transvaginal ultrasonography. Real-time shear wave elastography was used to measure Young’s modulus values, including the average Young’s modulus (Emean) and the maximum Young’s modulus (Emax) at 4 points: point A on the inner lip of the cervical os, point B on the outer lip of the cervical os, point C on the inner lip of the external os, and point D on the outer lip of the external os. Receiver operating characteristic (ROC) curve analysis was performed to compare the accuracy of Young’s modulus values at the 4 points, CL, and UCA in predicting SPB. Significant variables were used to construct a binary logistic regression model to predict the multifactorial predictive value of SPB, which was evaluated using an ROC curve. A total 176 valid cases, including 160 full-term pregnancies and 16 SPB, were included in this study. Receiver operating characteristic curve analysis revealed that Emean at point A, as well as Emean and Emax at point D, had a relatively high accuracy in diagnosing SPB, with area under the curve values of 0.704, 0.708, and 0.706, respectively followed by CL (0.670), SWE at point C (Emean 0.615, Emax 0.565), SWE at point B (Emean 0.577, Emax 0.584), and UCA (0.476). Binary logistic regression analysis showed that comorbidities during pregnancy (including diabetes mellitus, hypertension, cholestasis and thyroid dysfunction), CL, and Emean at point A were independent predictors of preterm birth. In addition, the AUC value of the logistic regression model’s ROC curve was 0.892 (95% CI: 0.804–0.981), with a sensitivity of 0.867, specificity of 0.792, and Youden’s index of 0.659, indicating that the regression model has good predictive ability for SPB. Real-time shear wave elastography showed a higher predictive value for SPB than CL and UCA. The SWE combined with CL and comorbidities during pregnancy model has a good predictive ability for SPB.

## 1. Introduction

Premature birth refers to delivery before 37 weeks of gestation and infants born during this period are referred to as premature neonates. Prematurity is the leading cause of neonatal mortality and long-term complications, with approximately 70% of the cases attributed to spontaneous preterm birth (SPB).^[1]^ In current clinical practice, transvaginal cervical length (CL) measurements are considered reliable and predictive tools for preterm birth and are frequently used for preterm birth surveillance. However, it fails to predict around approximately two-thirds of preterm births with only determination of CL.^[2]^ In obstetrics and gynecology, possible uses of cervical elastography include prediction of preterm delivery and successful labor induction according to the assessment of cervical stiffness Most pregnant women with a short cervix and no history of SPB can deliver at term without any intervention, and most preterm deliveries occur in pregnant women with a normal cervical length.^[3]^

Preterm birth may be the result of abnormal cervical remodeling,^[4]^ which can lead to SPB when the cervix softens prematurely, tissue hydration and compliance continue to increase, and cervical stiffness decreases.^[5]^ Ultrasound elastography allows quantitative assessment of tissue elasticity and stiffness,^[6]^ and elastography includes strain elastography (SE) and shear wave elastography (SWE).^[7]^ Real-time SWE is used to assess tissue structure by generating mechanical pulses through a probe, and shear wave propagation which leads to tissue displacement, leading to direct measurement of the shear wave velocity (m/s) or indirect measurement of the tissue Young’s modulus value, in (Kpa), to obtain the tissue stiffness value. This technique is applied to liver,^[8]^ thyroid,^[9]^ or breast diseases to assess tissue elasticity in different pathological states such as inflammation or malignancy.^[10]^ The application of elastic ultrasonography in obstetrics and gynecology has focused on the use of strain elastography in the diagnosis of uterine adenomyosis, uterine leiomyomas, endometriosis and cervical cancer.^[11]^

Yamaguchi et al^[12]^ investigated the feasibility of using tissue elastography to assess the pregnant cervix to identify women at high risk of preterm labor in 2007. Hernandez-Andrade et al^[13]^ found that cervical stiffness was more predictive of spontaneous preterm labor. Carlson et al^[6]^ demonstrated that before and after cervical ripening prior to delivery of a pregnant woman, the difference in SWE was statistically significant. Several recent studies have demonstrated the predictive value of cervical elasticity measured using the SWE technique for the occurrence of preterm labor.^[14]^ Furthermore, it has been reported that the uterocervical angle (UCA), which represents the angle between cervix and the anterior uterine wall, could serve as another potential marker for predicting SPB.^[15]^ Nevertheless, the predictive value of the UCA for SPB remains uncertain.

This study aimed to measure Young’s modulus values at different parts of the cervical canal using real-time shear wave elastography to predict the correlations between various indicators and SPB risk. Additionally, we aimed to compare the predictive value of these indicators with common ultrasound measurements such as CL and UCA. The predictive efficacy of individual factors as well as their combination for SPB was also analyzed in this study.

## 2. Materials and methods

### 2.1. Study design and participants

A total of 176 pregnant women between 16 and 36 weeks’ gestation who underwent antenatal examination at Baoji Central Hospital from June 2021 to December 2022 were included in the study, they were aged 21 to 44 years with an average age of 30.37 ± 4.29 years.

### 2.2. Inclusion and exclusion criteria

Inclusion criteria: All women with singleton pregnancies between 16 and 36 weeks gestation with regular antenatal checkups at Baoji Central Hospital and without fetal developmental abnormalities.

Exclusion criteria: Twin or multiple pregnancies; Excessive amniotic fluid, placenta previa, placental abruption, and vaginal bleeding during pregnancy; Previous cervical cone biopsy, LEEP knife, or cervical cerclage; and Termination of pregnancy due to maternal or fetal factors. This study was approved by the Ethics Committee of Baoji Central Hospital.

### 2.3. Ultrasonic assessment

**Figure 1.**
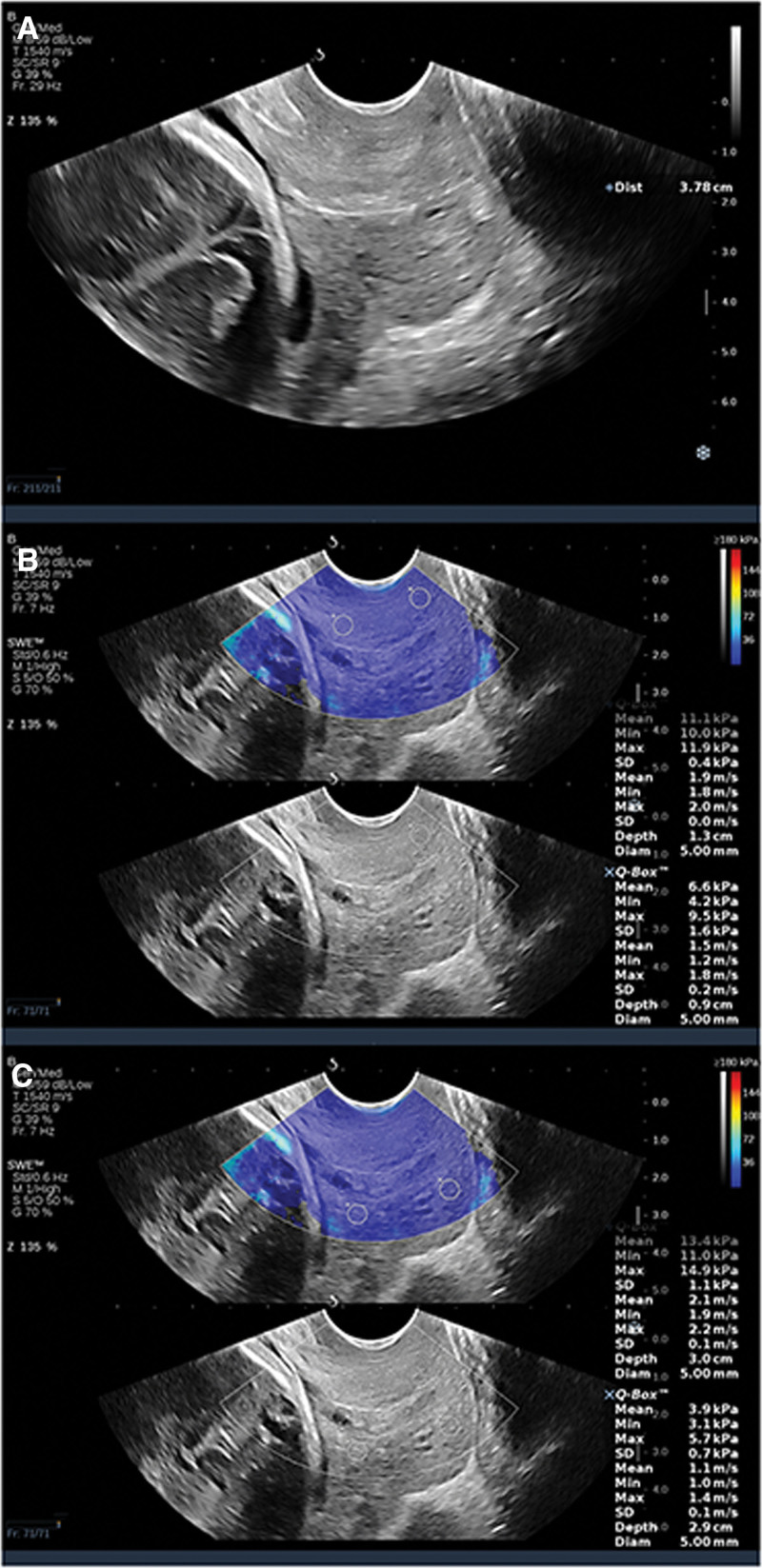
Images from a 26 year old woman in her 32nd week of pregnancy. (A) Cervical length measurement, the internal opening of the cervix is closed T-shaped. (B) Elasticity measurement of the internal and external foramina of the anterior labiate cervix. (C) Elasticity measurement of the internal and external foramina of the posterior labiate cervix. The pregnancy outcome was 38 + 1 weeks natural delivery.

**Figure 2.**
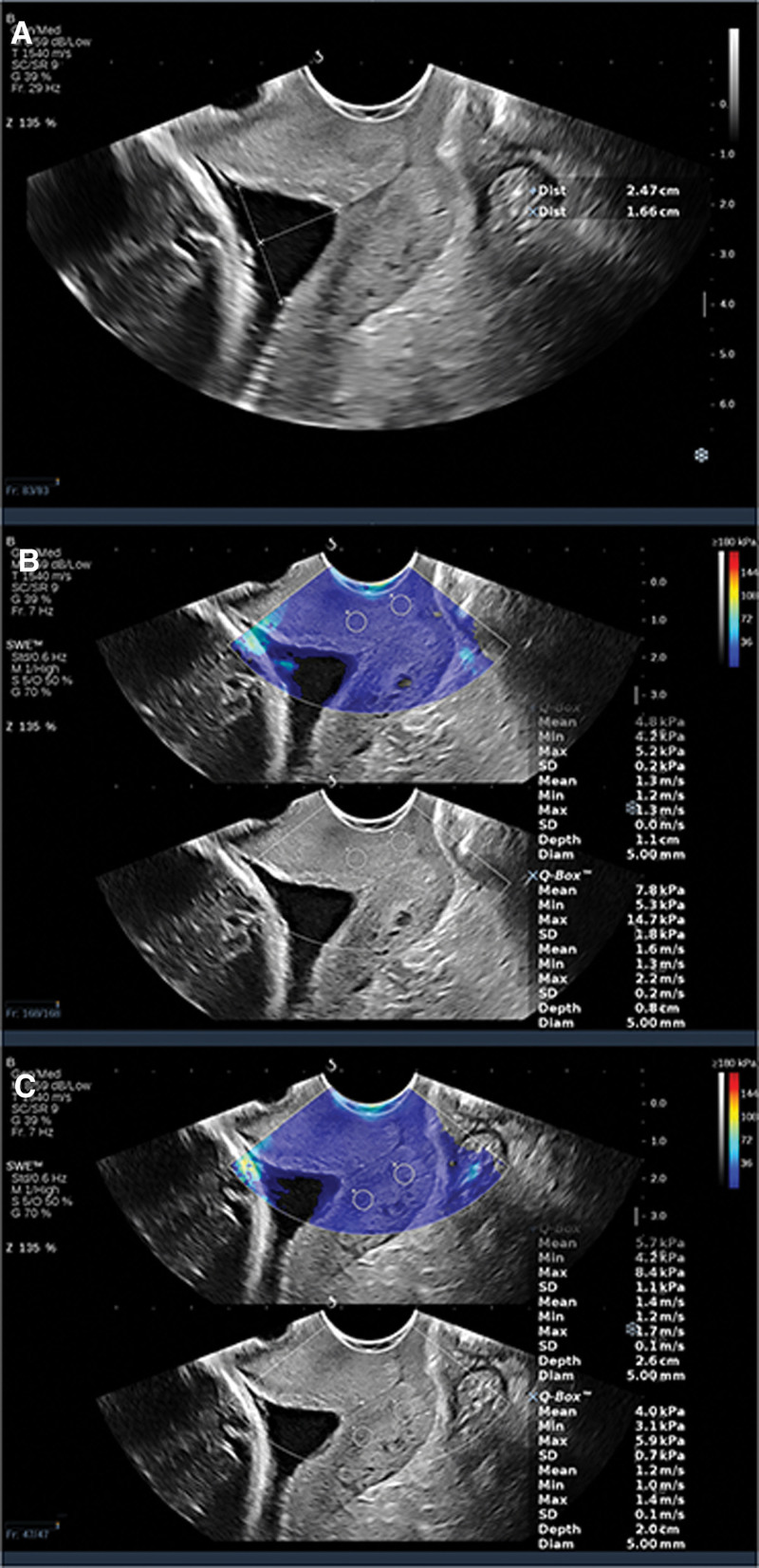
Images from a 31 year old woman in her 34nd week of pregnancy. (A) The cervix was shortened and the inner opening was U-shaped. The depth and width of the funnel were measured. (B) Elasticity measurement of the internal and external foramina of the anterior labiate cervix. (C) Elasticity measurement of the internal and external foramina of the posterior labiate cervix. The pregnancy outcome was premature rupture of membranes at 35 + 2 weeks.

The Aixplorer fully digital color Doppler ultrasound diagnostic instrument with super- high-speed shear wave elastography (SSI, Supersonic Imagine, France) was used. We conducted SSI testing on 175 pregnant women in weeks 16 to 36 of pregnancy and recorded the results. The steps were as follows Pregnant women were instructed to empty their bladder and lie on the examination bed in a supine position with their legs bent, exposing the external genitalia. The operator gently and slowly placed the probe into the vagina, without exerting pressure on the cervix. The probe was placed at the cervical os without squeezing the cervix to obtain a midsagittal view of the cervix for 2D and elasticity measurements. Measurements were taken 3 times and recorded: CL measurement: the distance from the internal os to the external os of the cervix. Cervical angle measurement: the angle formed by the line connecting the internal and external os of the cervix and the projection line of the internal os of the cervix with a distance of more than 3 cm from the internal os to the lower segment of the anterior wall.^[15]^ The SWE function was activated to cover the cervix with a blue sampling box. After the image was stabilized for 3 to 4 seconds, the region of interest size of the Q-BOX was adjusted to 5 mm. Four measurement points were selected: point A on the anterior lip of the internal os, point B on the anterior lip of the external os, point C on the posterior lip of the internal os, and point D on the posterior lip of the external os (see Figs. 1 and 2). The maximum Young’s modulus value (Emax) and the average Young’s modulus value (Emean) in Kpa, were recorded at each point of measurement point.

### 2.4. Statistical analysis

SPSS 20.0 (manufactured by MBI). was used to perform statistical analysis. Frequencies were used to represent categorical data. Differences between groups were analyzed using the chi-squared test or Fisher’s exact test. The Shapiro–Wilk test was used to test the normality of continuous data. Normally distributed data with homogeneity of variance were described as mean ± standard deviation (mean ± SD), and the independent samples *t* test was used for between-group comparisons. Non-normally distributed data or data with variance heterogeneity were statistically described as M(Q1, Q3), and the Mann–Whitney *U* test was used for comparison between groups. All tests were two-tailed, and the significance level was set at α = 0.05.

## 3. Results

### 3.1. General data of enrolled individuals

**Figure 3.**
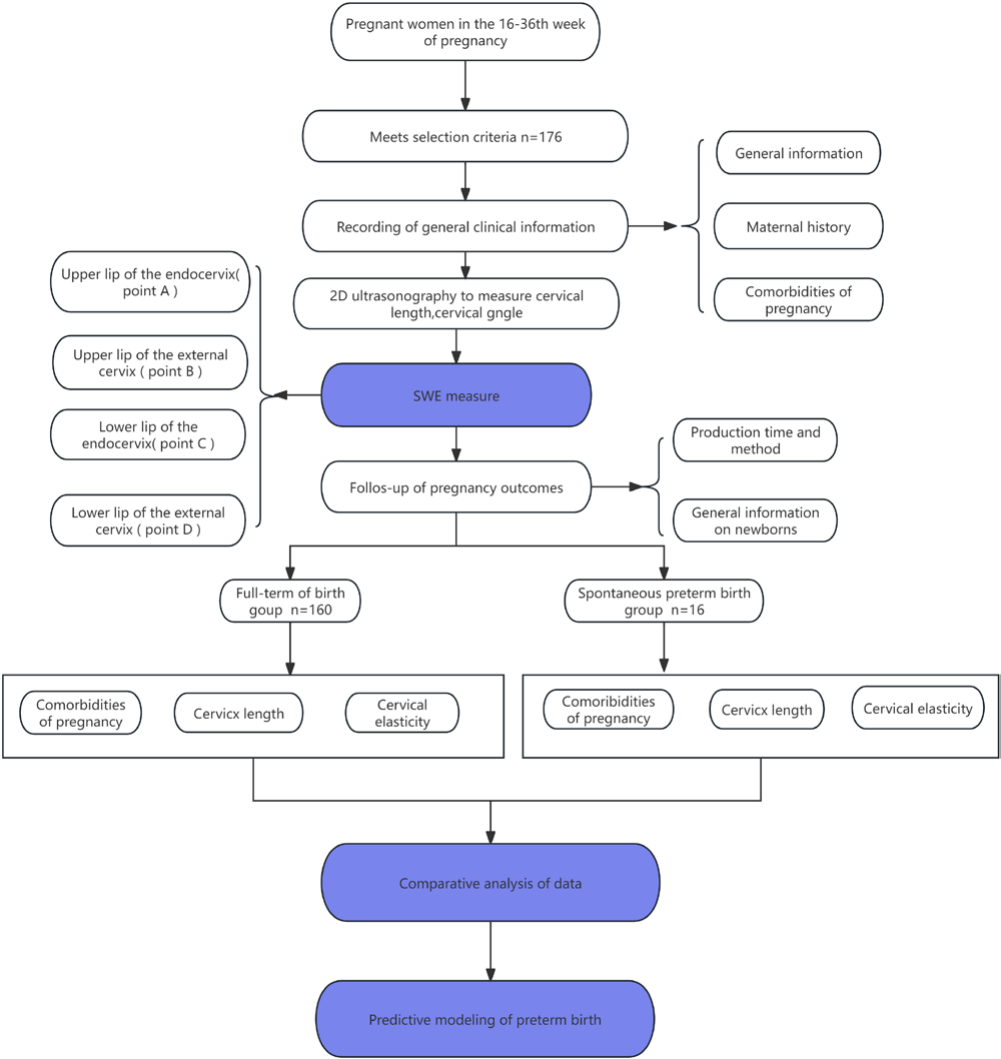
Statistical analysis flowchart.

A total of 176 valid cases were included in this study. The full-term pregnancy group consisted of 160 cases, aged 21 to 44 years, with a median age of 30 years. The SPB group consisted of 16 cases, aged 22 to 37 years, with a median age of 31 years. Based on the flowchart, analyze the factors affecting SPB in 176 cases (see Fig. 3).

The general characteristics of the 2 groups were compared (Table 1). In the SPB group, comprising 16 cases, the median delivery week was 36 weeks, with a mean CL value of 2.39 mm. Conversely, the full-term pregnancy group consisted of 160 cases with a median delivery week of 39 weeks and a mean CL value of 3.46 mm. The results showed No significant differences were observed in age, gestational age, age at menarche, number of pregnancies, number of live births, induced abortion, history of term births, or mode of delivery between the 2 groups (*P* > .05). However, there were significant differences between the 2 groups in terms of gestational age at delivery, pregnancy complications, and pregnancy outcomes (*P* < .05). There was a significant difference in weight, length, and 1-minute Apgar score between the 2 groups of newborns (*P* < .05), but no statistical difference in transfer to the NICU (*P* > .05). In the group of 16 cases of preterm birth, 7 patients had abnormal contractions and cervical shortening, and the presence of cervical insufficiency was clinically suspected. Analyzing the clinical data of the 16 patients with preterm delivery, 12 cases were found to have pregnancy complications, including 8 cases with gestational diabetes mellitus, 2 cases with gestational hypertension and 2 cases with cholestasis.

**Table 1:** Comparison of general data between the SPB group and the full-term pregnancy group.

	SPB (n = 16)	Full-term pregnancy (n = 160)	z*/t*/χ^2^	*P* value
Age	31 (27.5, 33.75)	30 (27.25, 33)	−0.501	.617
Gestational age at examination (wk)	26 (23, 30)	30 (26, 32)	−2.019	.043
Timing of menarche	13 (12.25, 14)	13 (12, 14)	−0.220	.826
Gestational age at delivery (wk)	36 (34, 37)	39 (38, 40)	−5.897	<.001
Number of pregnancies				
≤2 times	12 (75.0)	105 (65.6)	0.600	.438
2 times	4 (25.0)	55 (34.4)		
Number of live births				
0	13 (81.2)	101 (63.1)	2.747	.253
1	2 (12.5)	52 (32.5)		
2	1 (6.2)	7 (4.4)		
Pregnancy complications				
No	5 (31.2)	125 (78.1)	16.555	<.001
Yes	11 (68.8)	35 (21.9)		
History of induced abortion				
No	8 (50.0)	83 (51.9)	0.020	.886
Yes	8 (50.0)	77 (48.1)		
History of term births				
0	12 (75.0)	106 (66.2)	1.030	.598
1	3 (18.8)	48 (30.0)		
2	1 (6.2)	6 (3.8)		
Ever full-term pregnancy				
No	16 (100.0)	2 (1.3)	153.599	<.001
Yes	0	157 (98.7)		
Mode of delivery				
Vaginal delivery	8 (57.1)	81 (50.9)	0.198	.656
Caesarean delivery	6 (42.9)	78 (49.1)		
Newborn weight (g)	2833.00 ± 545.05	3333.38 ± 378.18	4.757	<.001
Length (cm)	49.5 (47.25,50)	50 (50,51)	−2.950	.003
1 minute Apgar score				
7	1 (6.7)	3 (2.2)	19.840	<.001
8	4 (26.7)	3 (2.2)		
9	10 (66.7)	131 (95.6)		
Transfer to neonatal intensive care				
No	15 (93.8)	136 (99.3)	2.009	.156
Yes	1 (6.3)	1 (0.7)		

SPB = spontaneous preterm birth.

### 3.2. Cervical characteristics

The CL, cervical morphology, cervical elasticity (elasticity values of points A, B, C, and D, including Emean and Emax), and cervical angle were compared between the preterm group and the term groups (Table 2). The results showed significant differences in cervical morphology between the full-term pregnancy group and the SPB group (*P* < .05). CL, Emean, and Emax of point A, Emean, and Emax of point C were significantly lower in the preterm group than in the term group (*P *< .05).

**Table 2:** Comparison of cervical characteristics between the SPB group and the full-term pregnancy group.

Cervical characteristics	SPB (n = 16)	Full-term pregnancy (n = 160)	*t*/χ^2^	*P* value
Cervical morphology				
T-shape completely closed	10 (62.5)	158 (98.8)	46.069	<.001
V-shape opened	5 (31.5)	1 (0.6)		
U-shape opening	1 (6.2)	1 (0.6)		
CL	2.39 ± 1.22	3.46 ± 0.59	−3.293	.001
Emean of A spot	8.76 ± 3.04	14.95 ± 8.21	−3.821	<.001
Emax of A spot	11.67 ± 4.19	18.73 ± 9.80	−3.482	<.001
Emean of B spot	7.86 ± 3.39	10.01 ± 6.58	−0.705	.481
Emax of B spot	11.35 ± 5.49	14.25 ± 10.25	−0.447	.655
Emean of C spot	10.53 ± 4.27	15.78 ± 8.51	−2.907	.004
Emax of C spot	16.12 ± 13.06	20.37 ± 10.52	−2.609	.009
Emean of D spot	5.79 ± 1.89	7.73 ± 3.82	−1.743	.081
Emax of D spot	7.54 ± 2.18	10.43 ± 5.49	−1.763	.078
UCA	97.64 ± 19.41	98.00 ± 23.04	−0.058	.953

CL = cervical length, Emax = maximum elastic modulus, Emean = mean elastic modulus, SPB = spontaneous preterm birth, UCA = uterocervical angle.

### 3.3. Predictive ability of cervical characteristics for SPB

The receiver operating characteristic (ROC) curves of the elasticity values of points A, B, C, D, CL, and UCA were plotted separately to compare the predictive ability of cervical characteristics for preterm birth. The results showed that the Emean of point A (AUC 0.704), Emean of point D (AUC 0.708), and Emax of point D (AUC 0.706) could independently predict preterm birth (Table 3).

**Table 3:** ROC analysis of the prediction for SPB.

	AUC	SE	*P*	95% CI lower	95% CI upper	Sensitivity	Specificity	Cutoff value
CL	0.670	0.094	.088	0.486	0.853	–	–	–
Emean of A spot	0.704	0.068	.040	0.570	0.838	0.605	0.778	11.350
Emax of A spot	0.665	0.073	.096	0.522	0.808	–	–	–
Emean of B spot	0.577	0.077	.440	0.426	0.727	–	–	–
Emax of B spot	0.584	0.071	.399	0.445	0.722	–	–	–
Emean of C spot	0.615	0.081	.248	0.457	0.773	–	–	–
Emax of C spot	0.565	0.099	.515	0.370	0.759	–	–	–
Emean of D spot	0.708	0.078	.037	0.555	0.861	0.829	0.556	4.750
Emax of D spot	0.706	0.072	.038	0.565	0.847	0.434	1.000	9.650
UCA	0.476	0.093	.811	0.293	0.659	–	–	–

CL = cervical length, Emax = maximum elastic modulus, Emean = mean elastic modulus, ROC = receiver operating characteristic curve, SPB = spontaneous preterm birth, UCA = uterocervical angle.

### 3.4. Construction of a multifactor preterm birth prediction model

**Figure 4.**
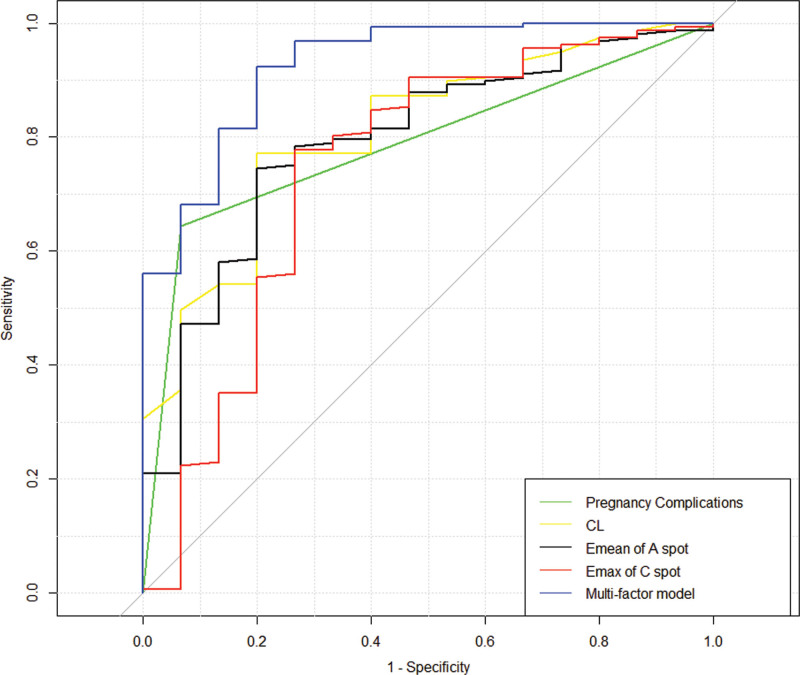
Receiver operating characteristic curve analysis. The multi-factor logistic regression model outperforms the single-factor model in predictive efficacy.

Using preterm birth as the dependent variable and variables with significant differences in the analysis of variance: gestational weeks, pregnancy complications, whether the pregnancy outcome was term, cervical morphology, CL, Emean, and Emax of point A, Emean, and Emax of point C as independent variables, a binary logistic regression model was constructed to predict the likelihood of preterm birth (Table 4). The backward stepwise method (Wald) was used for variable selection. The goodness of fit test (Hosmer and Lemeshow test chi-square = 7.603, *P* = .473) showed that the model had a good fit. Among them, pregnancy complications, CL, and Emean of point A were independent predictors of preterm birth (*P *< .05). The results showed that pregnancy complications increased the likelihood of preterm birth (B > 0, *P* < .05), while CL and Emean of point A were protective factors against preterm birth, indicating that higher values of CL and Emean of point A were associated with a lower risk of preterm birth (B < 0, *P* < .05). In addition, the AUC value of the logistic regression model’s ROC curve was 0.892 (95% CI: 0.804–0.981), with a sensitivity of 0.867, specificity of 0.792, and Youden’s index of 0.659, demonstrating the good predictive ability of the regression model for preterm birth. The multi-factor logistic regression model outperforms the single-factor model in predictive efficacy (see Fig. 4).

**Table 4:** Establishment of a multifactorial preterm birth model.

	B	SE	Wals	*P*	OR	95% CI lower	95% CI upper
Pregnancy complications	1.995	0.740	7.260	.007	7.353	1.723	31.386
CL	−1.824	0.547	11.110	.001	0.161	0.055	0.472
Emean of A spot	−0.199	0.098	4.148	.042	0.820	0.677	0.993
Emax of C spot	0.063	0.035	3.160	.075	1.065	0.994	1.141
Constant	3.337	1.530	4.759	.029	28.127		

*F* = 43.760, *P* < .001, *R*^2^ = 0.501.

CL = cervical length, Emax = maximum elastic modulus, Emean = mean elastic modulus.

## 4. Discussion

Innovative solutions to prevent preterm birth and hence reduce preterm birth rates worldwide are urgently requried. It has long been recognized measuring the CL using ultrasound can help predict SPB. However, CL measurement alone showed a 55% sensitivity in predicting preterm birth.^[16]^ Du, et al^[17]^ found that CL was not associated with sPTB at 11 to 14 weeks, 20 to 24 weeks, and 28 to 32 weeks of gestation. Changes in the collagen content and structure of cervical tissues during pregnancy lead to the physiological reconstruction of cervical tissues and changes in cervical tissue elasticity.^[18,19]^ Premature cervical changes are associated with preterm labor.^[20]^

SWE is cervical elastography provides information on cervical stiffness and can help predict preterm labor or successful induction of labor.^[21]^ In this study, the SWE technique was utilized for the ROC comparison of cervical elasticity values at 4 different points (A, B, C, and D), CL, and UCA to predict SPB. And the results showed that the AUC for Emean (A) was 0.704, followed by Emean (D), CL, Emean (C) and Emean (B). The elasticity value of point A at the anterior lip of the internal os of the cervix had higher sensitivity and specificity for predicting SPB than other indicators (sensitivity 0.605, specificity 0.778), but the diagnostic efficacy was still not satisfactory. However, the logistic regression model constructed from pregnancy complications, CL, Emean of point A, and Emean of point C significantly improved the predictive efficacy for preterm birth, with an AUC of 0.892 (95% CI: 0.804–0.981), sensitivity of 0.867, and specificity of 0.792.

Cervical hardness is determined by the collagen tissue. It was reported^[22]^ that the cervical collagen network has a high degree of heterogeneity, and the intensity of collagen protein along the cervical canal gradually decreases from the internal os to the external os. The collagen protein at the internal os was significantly stronger than that at the external os, and there were significant differences in the types of collagen proteins between the internal and external os. Therefore, the elasticity values at the 4 points of the internal and external os of the cervix were different. Our study found that the elasticity values gradually decreased from the internal os to the external os in both groups, and the preterm group had significantly lower elasticity values at the same positions compared to the term group. The elasticity value of point C at the lower lip of the internal os was higher than that of point A at the upper lip of the internal os, which is consistent with a previous study.^[23]^ This difference may be due to the influence of gravity on the amniotic fluid, resulting in higher measurement values at point C.

Peralta et al^[24]^ conducted cervical elastography at 4 different locations and found that the elasticity value at point A, the anterior lip of the internal os, was closely related to SPB. They also believed that cervical elasticity values had advantages over CL in predicting SPB. In this study, the elasticity value at point A of the upper lip of the internal os of the cervix had a larger area under the curve for predicting preterm birth, with a cutoff value of 11.3 kPa. When the average elasticity value at point A was below 11.3 kPa, the sensitivity for predicting preterm birth was 0.605 and the specificity was 0.778. The elasticity value at point D also had a high area under the curve for diagnosing preterm birth, but the sensitivity and specificity of the 2 indicators could not be met simultaneously, which may be due to the influence of the vaginal environment and the pressure of the vaginal probe on the measurement of the elasticity value at the external os. Hernandez-Andrade et al^[13]^ also reported that the elasticity value at the external os of the cervix was not associated with SPB.

In this study, the predictive value of CL for preterm birth was lower than that of cervical elasticity, which is consistent with previous studies. However, the morphological changes in the internal cervical orifice in this study were significantly different between the full term and preterm groups. The funnel is the shape of the internal uterine orifice, which is normally similar to a T shape. Funnel formation can be quantified by measuring the length and width of a funnel. Some studies have shown that using a funnel with a short CL could increase the predictive sensitivity of SPB from 61% to 74%^[25]^; However, from a clinical perspective, a funnel provides limited value and has minimal predictive value for PTB when used alone.^[26]^

Another important marker in the prediction of SPB is the Angle between the cervix and the anterior wall of the uterus.^[15]^ Women with SPB had a significantly higher mean UCA. In 1 study, UCA was assessed at term delivery in the first and second trimesters and showed a significant increase in UCA.^[27]^ However, LIobet et al^[27]^ reported a low UCA detection rate of 48.8%, these variable results were attributed to the small number of patients with SPB in each study, as well as the variable obtained measurements, gestational age, ethnicity, and previous history of the study population. In this study, UCA alone had the lowest diagnostic efficacy for preterm birth, which was similar to the results of LIobet’s study. In addition, this study found that pregnant women with a previous history of cesarean section may also contribute to their low diagnostic efficacy for preterm labor due to the adhesion of the uterine cesarean section incision site to the large abdominal omentum, which can result in increased cervical angle measurements.

However, a single screening factor is not ideal for the predicting efficacy of preterm birth. Recent findings by Xiaofeng Yang, et al^[28]^ suggested that combining cervical elasticity and length improves the prediction of SPB. Park et al^[29]^ showed that increasing cervical elastography improved the prediction of sPTD in women with CL shorter than 1.5 to 2.5 centimeters.

In this study, the combined evaluation of CL, elasticity values at point A and point C of the internal and external os of the cervix, and pregnancy complications showed good predictive ability for preterm birth. The area under the curve was the largest (AUC = 0.89) with good sensitivity and specificity. As maternal age and body mass index increase, the incidence of pregnancy complications also increases. Studies have shown that pregnancy complications such as hypertension, diabetes, and thyroid diseases have an impact on the occurrence of preterm birth.^[30]^ In our study, among the 16 preterm birth cases, here were 12 cases of co-morbidities in pregnancy, including 8 cases of gestational diabetes mellitus, 2 cases of gestational hypertension, and 2 cases of cholestasis in pregnancy. Pregnancy complications are important factors affecting pregnancy outcomes and severe cases may choose to terminate the pregnancy early, resulting in an increased rate of preterm birth due to the impact of pregnancy complications. A study has shown that combining CL with maternal medical history helps identify patients at risk for SPB.^[16]^ More patients with gestational diabetes mellitus were preterm deliveries in this study, suggesting that those with gestational diabetes mellitus may be at high risk for the occurrence of preterm labor.

In the study, we have obtained that cervical shear wave elastography has a predictive value for preterm labor. During routine obstetric ultrasound examination, we found that when the length of the cervix was less than 30 mm, we performed cervical elasticity shear wave measurements, and if we found that the cervical elasticity values were reduced, especially the elasticity value of the inner opening of the upper lip (point A) was lower than 11.3 Kpa, that is, the cervix of the patient was short and soft, which suggests that clinically there is the possibility of spontaneous preterm labor in the patient. This suggests the possibility of spontaneous preterm labor. This suggests the possibility of spontaneous preterm labor in this patient, and the clinic is instructed to take protective measures in advance, such as vaginal progesterone gel treatment or cervical cerclage, to prevent preterm labor from occurring. In addition, we found that the rate of preterm labor in patients with gestational diabetes mellitus was significantly higher than that in normal pregnant women, and patients with a short and soft cervix and a history of gestational diabetes mellitus should be a high incidence of preterm labor for us to focus on in the future.

There are many studies in the literature that confirm the predictive value of elastography in preterm labor. However, this technique has some limitations: firstly, the high complexity of the cervical microstructure and the attenuation of the shear wave have an impact on the measurement results; secondly, for nonlinear models, the elastic modulus and deformation are operator dependent.^[31]^ In addition, the viscoelasticity and nonlinear parameters of the cervix may be important due to the heterogeneity, various properties and viscosity of the cervical tissue. Measurement of cervical viscoelasticity is still at the stage of modeling in ex vivo.^[32]^ Valtorta et al^[33]^ proposed a new method to measure the complex shear modulus of soft biological tissues using a torsional resonator, and the measurement of soft tissue viscosity may yield more accurate operator-independent results, with possible future application to viscoelastic measurements of small, rounded, conical structures such as the cervix.

There are also some limitations existing in this paper. First, the small sample size and the relatively small number of cases of preterm birth may result in a lower evaluation of the predictive efficacy of a single factor for preterm birth, especially the lack of value in predicting SPB. In addition, this study spanned a wide range of gestational periods to increase the sample size. Thus, it is necessary to expand the sample size, accurately assess cervical elasticity at different stages of pregnancy, and objectively evaluate the various indicators for predicting preterm births.

In conclusion, this study measured cervical elasticity simultaneously with CL measurement and evaluation of cervical morphology, which is simple and easy to perform and could provide a more objective and accurate evaluation of SPB when combined with pregnancy complications that can be popularized and applied.

## Acknowledgments

The authors gratefully acknowledge financial supports.

The authors also acknowledge Dr Liujianxue and Zhangjing for help with language editing and figure artwork, and Dr Liujunjian for help with data analysis.

## Author contributions

**Conceptualization:** Huiling Lu.

**Data curation:** Huiling Lu, Yang Liu, Jiarui Qi.

**Formal analysis:** Huiling Lu, Yang Liu, Jiarui Qi.

**Investigation:** Fangrui Yang.

**Methodology:** Fangrui Yang, Dan Wu.

**Project administration:** Dan Wu.

**Supervision:** Mengli Hu.

**Validation:** Mengli Hu.

**Writing – original draft:** Huiling Lu, Yonghao Ji.

**Writing – review & editing:** Huiling Lu, Yonghao Ji.
